# Revealing the hidden structure of dynamic ecological networks

**DOI:** 10.1098/rsos.170251

**Published:** 2017-06-07

**Authors:** Vincent Miele, Catherine Matias

**Affiliations:** 1 Université de Lyon, 69000 Lyon; Université Lyon 1; CNRS, UMR5558, Laboratoire de Biométrie et Biologie Évolutive, 69622 Villeurbanne, France; 2 Laboratoire de Probabilités et Modèles Aléatoires, UMR CNRS 7599, Université Pierre et Marie Curie, Université Paris Diderot, Paris, France

**Keywords:** dynamic networks, network clustering, animal contact network, trophic network, stochastic block model

## Abstract

In ecology, recent technological advances and long-term data studies now provide longitudinal interaction data (e.g. between individuals or species). Most often, time is the parameter along which interactions evolve but any other one-dimensional gradient (temperature, altitude, depth, humidity, etc.) can be considered. These data can be modelled through a sequence of different snapshots of an evolving ecological network, i.e. a dynamic network. Here, we present how the dynamic stochastic block model approach developed by Matias & Miele (Matias & Miele In press *J. R. Stat. Soc. B* (doi:10.1111/rssb.12200)) can capture the complexity and dynamics of these networks. First, we analyse a dynamic contact network of ants and we observe a clear high-level assembly with some variations in time at the individual level. Second, we explore the structure of a food web evolving during a year and we detect a stable predator–prey organization but also seasonal differences in the prey assemblage. Our approach, based on a rigorous statistical method implemented in the R package dynsbm, can pave the way for exploration of evolving ecological networks.

## 1. Background

Networks are widely used in ecology as they provide a powerful tool for modelling the complex interplay between ecological entities [1]. Depending on the context, those entities can be different species or different individuals while their interplay may be as diverse as trophic, competitive, cooperative relations or even contacts measured through physical proximity. Studying these networks can help in answering important ecological questions, for example, about the structure of these interactions and their robustness to external factors.

As Newman & Leicht [2] pointed out about 10 years ago ‘much of the current research on networks [...] aimed at answering the question: how can we tell what a network looks like, when we can’t actually look at it?’ As a first response, the solution was to develop and use descriptive statistics and network measures such as connectance or centrality (see [3] for a comprehensive list). This approach considers any ecological network as a whole, assuming that the network is homogeneous. Another solution was to go beyond descriptive statistics and consider ‘network clustering’, i.e. grouping entities according to their common properties. This technique addresses fundamental questions about any underlying network structure: Is there any peculiar non-random mixing of entities that would be a sign for a structural organization [4]? Is there, for instance, any compartmentalization [5], hierarchical organization [6] or nestedness [7]?

Nowadays, recent technological advances (sensors, GPS technology, etc.) and long-term data studies have given rise to an avalanche of temporal data that need to be appropriately modelled. Data acquired over time can be aggregated within relevant time intervals (days, seasons, years, etc.) and consequently produce snapshots of a same ecological network at different time steps. With these new data, one can potentially address new ecological questions which might not be tackled through the analysis of the ‘static’ network where data are aggregated over full recording time. In the same way, snapshots of an ecological network along any one-dimensional factor (such as temperature, altitude, depth, humidity, etc.) may help analyse the evolution of the network structure along this gradient [8]. However, addressing those new questions requires the development of new methodological tools. Up to now, very few proposals have been made to handle what we call here ‘dynamic networks’, namely any sequence of snapshots of a same ecological network along a one-dimensional parameter that we most often call ‘time’ (see also [9] for a review about multilayer networks).

We proposed a novel network clustering approach to analyse dynamic networks [10]. This approach mainly consists in extending one of the techniques dedicated to find structural patterns in static networks, now focusing on their dynamics. In this paper, we aim at showing how this method can be appropriate to analyse real ecological datasets. After stating the key concepts and introducing the terminology used for handling dynamic networks, we will analyse the dynamic contact network in a colony of ants [11]. Contact networks represent a relevant proxy to study animal sociality [12]. In the literature, these networks may be built from field observations of associations between animals (e.g. giraffes in [13]), trapping data (e.g. field voles in [14]) and more recently and predominantly from sensor-based measurements (e.g. sharks in [15]). These data are now available for large time periods, ranging from days to years of observations for instance. It is therefore possible to investigate the (in-) stability of the social structure [16] and potentially question the impact of other time-related factors (seasonal changes, response to stresses such as draught, arrival/departure of a particular individual, etc.). Then we will present the study of a seasonal trophic network (or *food web* [17]). The structure of trophic relations has been intensively studied in the network framework (see [18] for a clustering perspective). Nowadays, following the seminal work of [19], new datasets allow for monitoring the variation of this structure along temporal gradients (seasons or years), spatial gradients (latitudinal or longitudinal for instance [20]) or qualitative gradients (increasing habitat modification [21]). We will focus only on dynamic trophic networks corresponding to different temporal snapshots of a food web. In this context, studying such structural variation (or on the contrary, structural stability) can be appropriate to analyse the system’s response to major changes (species extinctions, environmental perturbations, climate change, etc.).

The two fundamental questions we will focus on here are the following: Are there any relevant statistical patterns in the dynamic network? If so, how does this structure vary with time (or along the sequence)? In this article, we answer these two key points and argue that this is a first stage in further understanding and predicting processes on dynamic ecological networks such as event spreading (infection [22] or extinction, for instance).

## 2. Material and methods

### 2.1. From static to dynamic networks

**Figure 1.**
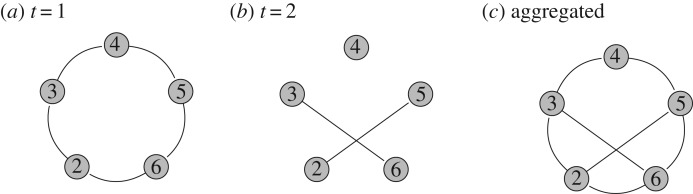
Same data (*a*,*b*) modelled by a two time steps dynamic network or (*c*) aggregated over the whole time period into a static network. The structure in the static case does not reflect the complexity of the network structure which clearly varies with time. Indeed, edges present at *t*=1 and *t*=2 are disjoint.

An ecological network is composed of nodes that correspond to any ecological entities (e.g. species, individuals or communities), while edges (or links) characterize the presence/absence of an interaction between any two entities and may be valued in some cases. For instance, values may be the frequencies of contacts between two individuals [23] or the number of field observations of interactions between two species. When this network is unique and covers an entire time period, it is called a *static* network. While many empirical data were aggregated over a whole period of observation recording, it is important to realize that such aggregation could lead to an incorrect understanding of the network structure due to the smoothing aggregation process (cf. figure 1). An approach to study the temporal dynamics of a set of interactions is the *discrete time snapshots* approach (see [24] for a complete perspective). It consists of aggregating data over specific time frames (days, months, seasons, years or any relevant frame regarding the ecological system of interest) and to obtain what Blonder *et al.* call *time-aggregated dynamic networks* [24]. In the following, we use the term *dynamic networks* and while we refer to time as being the parameter that drives the evolution, we recall that this could be any other relevant one-dimensional factor.

Formally, we assume *T* time steps, a number *N*_*t*_ of nodes at each time step *t*, a total number of nodes *N* (with *N*<*N*_1_+⋯+*N*_*t*_) and edges record the presence (possibly valued) or absence of an interaction between any two pairs of nodes at each time step. Note that our set-up is different from the one corresponding to the observation of the full interaction flow, namely when data consist of the complete knowledge of edge appearance and disappearance along a continuous gradient. Indeed in our case data are still aggregated over some time intervals or correspond to a sequence of networks which are specific to a set of discrete values of a one-dimensional factor. When considering continuous time interaction flow data, the object of interest (the flow) is called a *temporal network* [25] and this set-up will not be explored in this article.

Lastly, it is important to mention that the time frame selection may be an issue in cases where choosing the resolution for the time aggregation is not driven by the ecological question. Indeed, in many cases, the choice of the time frame is expert-based: for instance the dataset from [26] consists of *T*=52 days of observation including the breeding season, but it is possible to restrict to *T*=3 networks (before, during and after the breeding season) to study the network variations due to reproduction period. This choice might not be harmless and for instance [24] showed that the degree distribution in networks can be sensitive to the time frame selection (see also [27] for a statistical perspective). It is out of the scope of the present work to explore this frame selection problem.

### 2.2. Stochastic block models

**Figure 2.**
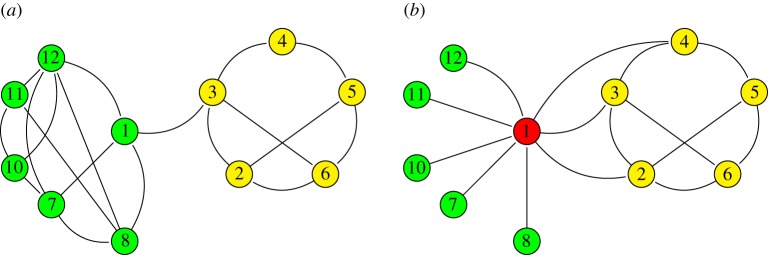
(*a*) Network with a clear modular structure with two modules (green and yellow). (*b*) Network with a complex structure including different patterns: a module (yellow), a hub (red) and a set of peripheral nodes (green). Both networks can be modelled by a SBM with different parameters that capture the structural organization, with two and three groups, respectively. Besides, it is not clear what would be the results of a modular detection algorithm on the second network (as it is not modular).

In the field of network analysis, one of the most exciting research problems of the last decade has been the network clustering question. Moving beyond descriptive statistics, the goal here is to propose algorithms to extract a high-level view of complex networks, i.e. zooming out the network. Network clustering consists of grouping nodes based on their common characteristics. It often rhymes with finding *modules* or *communities* (or *compartments* [28]). A module is a set of nodes with much more edges between these nodes than with the others. An important drawback of module-based approaches appears when, quoting Newman & Leicht [2], we ask: ‘could there be interesting and relevant structural features of networks that we have failed to find simply because we haven’t thought to measure the right thing?’ In other words, is it relevant to search for modular structure in a network that can be structured in any other ways? Following this objection, methods based on statistical inference arose which rely on the principle of grouping nodes that have similar interaction patterns (e.g. hubs, modules, peripheral nodes; figure 2) without any *a priori* knowledge. This is the purpose of a general class of models called *stochastic block models* (SBM).

SBM have been developed for analysing complex networks [2,29–32] and more recently used to decipher the structure of ecological networks such as hosts–parasites [33], food webs [34,35] and multi-interactions network [4].

Let {*Y*_*ij*_}_1≤*i*,*j*≤*N*_ be random variables modelling the presence/absence of edges between any possible couple of nodes (*i*,*j*). The model may be defined either for directed or undirected networks and may allow the presence of self-interactions (edges *Y*_*ii*_). The principle of SBM is the following: we assume that the ecological entities (nodes) can be gathered into *Q* groups based on their common interaction properties. Therefore, the distribution of *Y*_*ij*_ is specified conditionally on the group memberships such that

$$Y_{ij}∼f(\Theta_{ql})\;|\;i\in \text{group }q,\;j\in \text{group }l,$$

where *f* is any probability distribution parametrized by *Θ* (called interaction parameter). The group memberships are unknown, as well as the interaction parameters. An EM-like algorithm (expectation–maximization [36]) allows for jointly estimating memberships and parameters [30]. The statistical procedure finally displays a high-level view of the network: what kind of interaction patterns are present (through the interaction parameters *Θ*_*ql*_) and which nodes participate in those patterns (through the group memberships).

A key advantage of SBM is the possibility to plug any probability distribution *f* in order to fit any kind of interactions. For instance, one can use a Bernoulli distribution for binary interactions [29], a Gaussian for frequencies or a Poisson for a number of interactions (weighted interactions [33]), a multinomial for finitely many values or even a multivariate distribution for multivariate edges. One can also use the combination of any of those distributions with a Dirac mass at 0 so as to obtain a 0-inflated distribution that accounts for sparsity in the network (i.e. a distribution that allows for frequent 0 values). Relying on a probabilistic framework allows for modelling some randomness and variability in the observations and consequently provides robustness to possible errors or missing data.

### 2.3. Dynamic stochastic block models

How can we analyse dynamic ecological networks to extract structural information? At the time of writing, only a few alternatives based on descriptive statistics [25] or on evolving modules [9,37] have been considered. Following the above mentioned objections, we claim that a model-based clustering approach could be relevant and we proposed to extend the SBM approach to dynamic networks introducing *dynamic stochastic block models* (dynSBM) [10]. An important question to ask is what could be the meaning of zooming out an object that can change with time? Our answer is to capture the dynamics of a high-level view of the network. This means tracking the evolution of the group behaviours (i.e. the interaction parameters) as well as the node group memberships with time.

Technically, dynSBM relies on a collection of SBM for modelling the different snapshots at each time step combined with *N* (the number of nodes) independent and identically distributed Markov chains that capture through time the evolution of the group a node belongs to. A consequence of this modelling is that at any time step *t*, the estimate of the group of a node *i* depends on the groups of the other nodes and on the groups of this same node at other times. The model is now characterized through

$$\begin{array}{rl} & Y_{ij}^{t}∼f(\Theta_{ql}^{t})\;|\;i\in \text{group }q,\;j\in \text{group }l\text{ at time }t\\ & P(i\in \text{group }q\text{ at time }t\;|\;i\in \text{group }q^{\prime }\text{ at time }t-1)=\Pi_{qq^{\prime }} \end{array}$$

where *Π* is the (common) transition matrix of the *N* different group membership Markov chains. Reconstructing the different SBM and the common Markov chain parameters has to be done jointly, looping over the two following steps until convergence: (i) updating group membership using (*Θ*, *Π*) jointly and (ii) updating *Θ* and *Π* using group membership.

We stress that the dynSBM approach is different from a naive one that would separately cluster each network and use an ad hoc procedure to resolve the label switching problem between two time steps. Indeed, the Markov chain modelling induces dependencies between the networks at different time steps. As a consequence, the groups recovered for one specific network use information about the others. We also mention that the (maximal) number of groups is fixed with time though some groups might be empty at some time steps. This number may be selected either through a statistical model selection criterion called ICL (integrated classification likelihood) or relying on heuristic procedures (see [10] for a complete discussion about this issue).

It is also important to mention that the dynSBM is one of the rare tools that can deal with arrivals and departures of entities (corresponding either to species invasion/extinction or to birth/arrival/death/departure of individuals) through the possible presence or absence of nodes at the different time steps.

To summarize, the dynSBM approach allows for exploring the following questions: (i) Is there any high-level structure in the network, i.e. does dynSBM find more than a single group of nodes? (ii) Does this network structure vary with time, i.e. are the node group memberships evolving with time? (iii) What are the group switches trends and frequencies, i.e. what are the values of the underlying Markov chain parameters? (iv) Are there any stable or unstable individuals, i.e. are there peculiar group membership trajectories?

### 2.4. Datasets

#### 2.4.1. Colonies of the ant *Camponotus fellah*

Colonies of the ant *Camponotus fellah* were followed with a tracking system that monitored the individual positions over days of observations and dynamic social interactions were deduced from physical proximity [11]. The data correspond to a colony of *N*=152 *Camponotus fellah* ants observed during *T*=10 days. Edges of the resulting dynamic network are weighted by the number of interactions between each pair of ants and the network is thus undirected with no self-interactions.

#### 2.4.2. Macro-invertebrate community of Broadstone Stream

This dataset concerns the aquatic macro-invertebrate community of Broadstone Stream in southeast England [17]. Six seasonal connectance food webs were recorded, one every two months from May 1996 to April 1997. We restricted here to simple presence/absence information on species (nodes) and binary feeding links (edges) and did not consider quantitative data. The number of sampled species of this aquatic macro-invertebrate community varies seasonally (up to *N*=26 in total including 10 predators) as well as the number of directed links. This dataset forms a dynamic trophic network with *T*=6 snapshots (May, August, October, December 1996, February, April 1997).

## 3. Results

### 3.1. Dynamic contact network of ants

After examination of the weights distribution, we chose to bin the edge weights into *M*=3 categories corresponding to *low, medium* and *high* interaction intensity. We consequently fitted a dynSBM with a multinomial distribution *f* (in fact as many multinomials as the number of group pairs {*q*,*l*}). We selected *Q*=3 groups with the heuristic ‘elbow’ method (which consists of finding the point where the slope of the model log-likelihood significantly decreases, see [10]).

**Figure 3.**
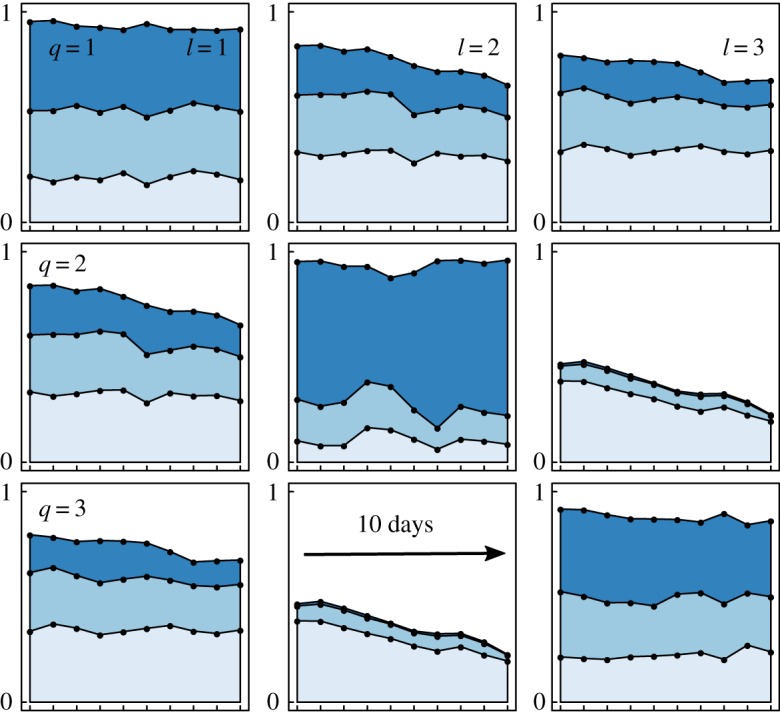
Interaction properties between groups on the ants dataset. Interaction presence and intensity between nodes in any of the *Q*=3 groups to the others are represented in a global *Q*×*Q* matrix; each cell contains *T*=10 time points on the *x*-axis corresponding to the different time steps. Each square represents four areas: the white area is the proportion of absent edges among all possible pairs of interactions; light to dark blue areas correspond to the proportion of edges (among present ones) with low to high intensity value, respectively. Plot obtained with the connectivity.plot function of the dynsbm package.

We first focus on the overall structure of the dynamic network by observing the inter/intra-group interaction properties, as shown in the different cells of figure 3. Note that the global *Q*×*Q* matrix shown here is symmetric as the network under consideration is undirected. The first key concept here is the *sparsity* level, i.e. the amount of edges that are present over all the possible relations (without considering edge values). We clearly see that intra-group interactions are very frequent, in particular in groups 1 and 2 where almost any pairs of ants of these groups are in contact (figure 3, large blue areas in diagonal plots). This pattern is stable in time (10 days, *x*-axis in figure 3). The most interesting trend about inter-group interaction concerns group 3 which contains ants that interact with those of group 1 but much less with those of group 2 (figure 3, smaller blue areas in cells (2,3) or (3,2) than in cells (1,3) or (3,1)). These properties are the key factor determining the group boundaries (i.e. the memberships) as the other inter-group interactions remain frequent. The next key notion is the *intensity* level that focuses on the values of present edges and reflects the point to which ants of two given groups are more likely to be in contact with low to high intensity. Interestingly, when two ants of group 2 are in contact (edge is present), they are likely to be in contact with a high intensity/frequency value (figure 3, large dark blue area in cell (2,2)). On the contrary, even if some contacts exist between these ants and those of group 3, which is already unusual, these sporadic contacts exhibit low intensity (figure 3, larger light blue area in cell (2,3) or (3,2)). Here, the remaining intra/inter-group intensity levels do not reveal any other interesting pattern (equal proportion of intensity categories). With all these observations, we deduce that group 2 is a so-called module (highly intra-group connected ants) relatively disconnected from group 3 and that group 1 gathers ants ‘at the interface’, i.e. interacting with partners from any of the three groups.

**Figure 4.**
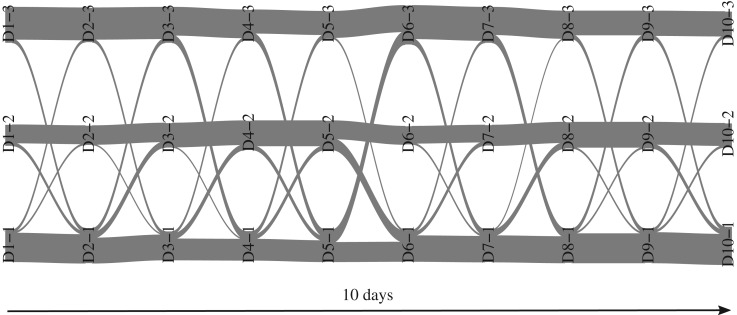
Alluvial plot showing the dynamics of the group memberships on the ants dataset. Between 2 days (*t*=1,…,10 on the *x*-axis), each line is a flux that represents the switch of one or more ants from a group to another group (*q*=1,…,3 represented on the *y*-axis). Here, D*t*–*q* denotes group *q* from day *t*. The thickness of each line is proportional to the corresponding counts. Plot obtained with the alluvial.plot function of the dynsbm package.

We now investigate whether there are some interesting trends in the turnover of group membership. In other words, we wonder whether all ants have the same propensity to move from one group to another one. We first observe that the global group turnover (i.e. the amount of group switches) is low: 46% of ants never switch group. Moreover, there are no group switches between groups 2 and 3 (figure 4, no fluxes between these groups over time). This observation, along with the low level of interactions between group 2 and 3 that we discussed before, suggests the existence of a ‘barrier’ between these groups that could be a consequence of space positioning. Indeed, Mersch *et al.* [11] showed that ants were distributed over three social groups (obtained by analysing each daily static network and combining those analyses) with different interaction patterns and that there existed some spatial segregation of the groups. We thus propose to compare our groups obtained with dynSBM (which are evolving with time) and the social groups of Mersch *et al.* (which are fixed with time). Focusing on the ants that stay in the same group at least 8 days over 10 (111 ants over *N*=152), we note a quasi-perfect match between Mersch *et al.* groups and our groups (table 1). The modular group 2 corresponds to the *foragers* of Mersch *et al.*, while the other groups 1 and 3 correspond to the *cleaners* and *nurses*, respectively. Besides retrieving this functional group, we provide another relevant information: it is now possible to study ants playing different social roles over time, i.e. those that experience group switches at certain time points. Indeed, the dynSBM groups allow one to pinpoint these interesting individuals that modified their behaviour over time and that can be of particular interest for specialists of the *Camponotus fellah* system.

**Table 1.** Contingency table between Mersch *et al.* [11] functional groups and our dynSBM groups (only ants staying at least 8 over 10 days in the same group are considered here; 75% of the total number of ants).

	cleaners	foragers	nurses
dynsSBM group 1	29	1	4
dynsSBM group 2	2	29	0
dynsSBM group 3	0	0	42

### 3.2. Broadstone Stream seasonal food webs

Five species were not sampled each month but this situation where nodes are present/absent over time is supported by the dynSBM model. It is also important to mention that self-interactions (cannibalism) exist for six out of 10 predator species. Again, the model allows for this behaviour which might distinguish predators among them and structure the network. We fitted a dynSBM with Bernoulli distributions *f* and we selected *Q*=4 groups with the ICL criterion (which is a penalized-likelihood criterion that is well established in the model-based clustering framework [10]).

**Figure 5.**
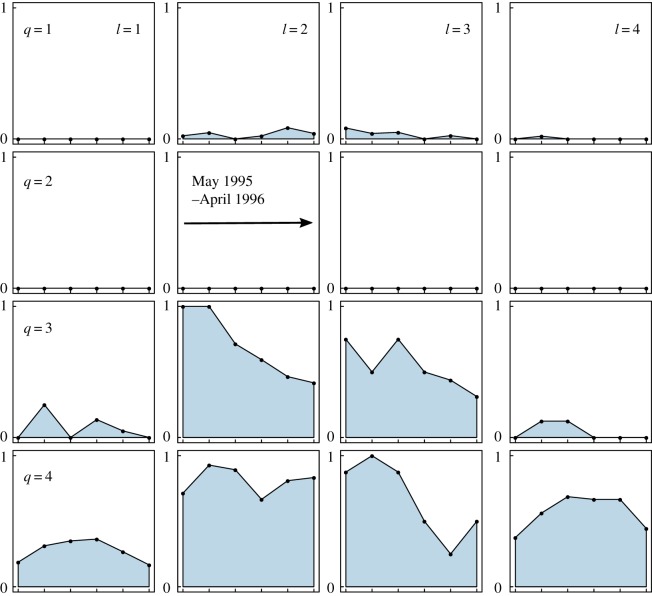
Interaction properties between groups on the food web dataset. Same as figure 3 for *Q*=4 groups and *T*=6 time steps. In this case, only interaction presence is shown (blue area) as we consider binary edges. Moreover, the *Q*×*Q* matrix shows directed interaction from group *q* (rows) to group *l* (columns).

The inter/intra-group interaction properties shown in figure 5 are not symmetric because we consider directed networks. Therefore, for each pair of groups {*q*,*l*} their interaction characteristics are twofold: how often species of group *q* eat those of group *l*, and the reverse. As such, group 4 is composed of *omnivorous* species that eat species of any other groups, but are only eaten by species of their own group (this includes cannibalism). Group 3 has the same properties as group 4 with a significant difference: species of group 3 do not eat those of group 4. We conclude that species from group 3 occupy intermediate positions in food chains whereas those of group 4 are top predators. Indeed, group 4 is mainly composed of the three largest species (the top predators *Cordulegaster boltonii*, *Sialis fuliginosa* and *Plectrocnemia conspersa*), whereas group 3 contains mostly three small species (the larvae of the tanypod midges *Macropelopia nebulosa*, *Trissopelopia longimana* and *Zavrelimyia barbatipes*). Group 2 overall gathers preys that are mostly eaten by predators of groups 3 and 4. Species from group 1 are ‘hidden’ species: they do not eat much, and are not much eaten either. This group is built on a statistical argument (fewer links from/to other species compared to species of the other groups), therefore, it is not coherent from a taxonomic point of view as it gathers a mixture of predators with little activity and secondary preys that we call *peripheral species*. Lastly, the results reflect the overall decrease in the number of links after October (figure 5, blue area decreasing with time in boxes) which is partly due to the fact that tanypods become less predatory and more detritivorous after autumn [17].

**Figure 6.**
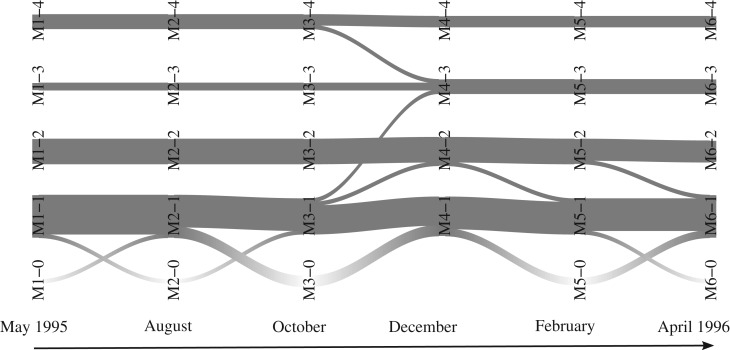
Alluvial plot showing the dynamics of the group memberships on the food web dataset. Same as figure 4 for months t=1,…,6. Here, M*t*–*q* denotes group *q* on month *t* for *q*=0,…,4 where special group 0 gathers absent entities at each time step.

Now, we explore whether species positions in the food chains (namely being top or intermediate predators, peripheral species or common preys) evolve or stay constant across the seasons. We do not expect a low-level prey to become a top predator, but the group boundaries may change due to seasonal diet variations; for instance, *Macropelopia nebulosa* eats lots of *Nemurella pictetii* in August but not in April as this species becomes too large [17]. Figure 6 shows that group memberships remain stable before winter, but some changes are observed between October and December. In particular, the tanypod species *Macropelopia nebulosa* belongs to group 4 and changes to group 3 in winter. Indeed, in summer and autumn only, this species diet is similar to the one of the other members of group 4 (the three competitive top predators, that eat each other) while also being their prey. Still between October and December, the stonefly *Siphonoperla torrentium* becomes an active predator (with only 1 prey in October and 5 in December) and moves from group 1 to group 3. The prey *Prodiamesa olivacea* becomes commonly eaten during winter and is consequently integrated into group 2 during this period (and moves back to group 1 in April). Lastly, we observe that *Brillia modesta* changes from group 2 to group 1 between December and February: this species becomes the exclusive prey of the top predators during this period, whereas it is a common prey during the other months.

## 4. Discussion

The inclusion of time in network analysis has been a recent challenge that requires ad hoc modelling approaches. The success of these approaches has to be measured by their ability to extract substantial additional information that would not be caught by a traditional static network analysis. To this aim, we evaluated the dynamic stochastic block model as a new candidate to decipher temporal trends or temporary patterns in dynamic ecological networks.

On the ants interaction network dataset, while the overall group behaviour trends are captured by the model, different individual behaviours are also highlighted. This way, our results can be interpreted at different scales. On the food web dataset, the model underlines a clear trophic organization but also seasonal differences in the prey assemblage. These results require further investigation by experts, but it is interesting to note that our approach can play a key role in extracting unexpected patterns.

Our analysis is grounded on a rigorous statistical method and can be reproduced on other datasets with the R/C++ package dynsbm that can handle hundreds to thousands of nodes. It is hence one of the very first tools for ecologists facing the recent availability of time-ordered datasets or that would like to explore the evolution of ecological networks with respect to a one-dimensional factor.

## Data accessibility

The ants dataset [11] is available at http://datadryad.org/resource/doi:10.5061/dryad.8d8h7. The food web dataset is available in plain text in table 2 of [17]. Both datasets are also available as R data objects in the R software package dynsbm which is available at http://lbbe.univ-lyon1.fr/dynsbm and from the CRAN repository. Code to reproduce the data analysis is available in the R help associated to each R data object.
